# 106. Risk Classification to Differentiate Autoimmune from Viral Encephalitis

**DOI:** 10.1093/ofid/ofab466.106

**Published:** 2021-12-04

**Authors:** Alejandro Granillo, Michael Hansen, Mohammed S Samannodi, Rodrigo Hasbun, Rodrigo Hasbun

**Affiliations:** 1 University of Texas Health Science Center at Houston, Houston, Texas; 2 Baylor College of Medicine, Houston, TX; 3 Umm Al-Qura University, Mecca, Makkah, Saudi Arabia; 4 University of Texas Health Science Center, Bellaire, TX

## Abstract

**Background:**

Autoimmune encephalitis is an urgent treatable etiology that needs to be differentiated from viral encephalitis. Prompt recognition and therapy is of utmost importance.

**Methods:**

We performed a retrospective cohort of encephalitis cases in 16 hospitals in Houston, Texas, between January 2005 and December 2019.

**Results:**

A total of 1,310 adult (age ≥18 years) inpatient hospital admissions were identified by the presence of an encephalitis-related discharge diagnosis per the International Classification of Disease 9^th^ edition codes. Of these, only 279 cases met the 2013 International Encephalitis Consortium criteria for probable encephalitis. A laboratory confirmed diagnosis of autoimmune encephalitis or viral encephalitis was identified in 36 (12.9%) and 88 (31.5%) cases, respectively. There were 155 cases (55.5%) that had no identifiable cause and were considered idiopathic.

As compared to viral encephalitis, patients with autoimmune encephalitis were more likely to be younger (< 60 years old), have a subacute (6-30 days) or chronic ( >30 days) presentation, have seizures, and have psychiatric and/or memory complaints (P< 0.001). Furthermore, patients with autoimmune encephalitis were less likely to be febrile and to lack inflammatory cerebrospinal fluid (CSF) (defined as white blood cells < 50 per microliter or protein < 50 milligrams per deciliter) [See Table 1]. In the multivariable logistic regression model, subacute/chronic presentation, psychiatric and/or memory complaints, and lack of inflammatory CSF were significantly associated with autoimmune encephalitis. Using these 3 variables, patients were classified into 3 risk categories for autoimmune encephalitis: low risk (0-1 variables); 0%; intermediate risk (2 variables); 16%; and high risk (3 variables); 83% (P value < 0.001).

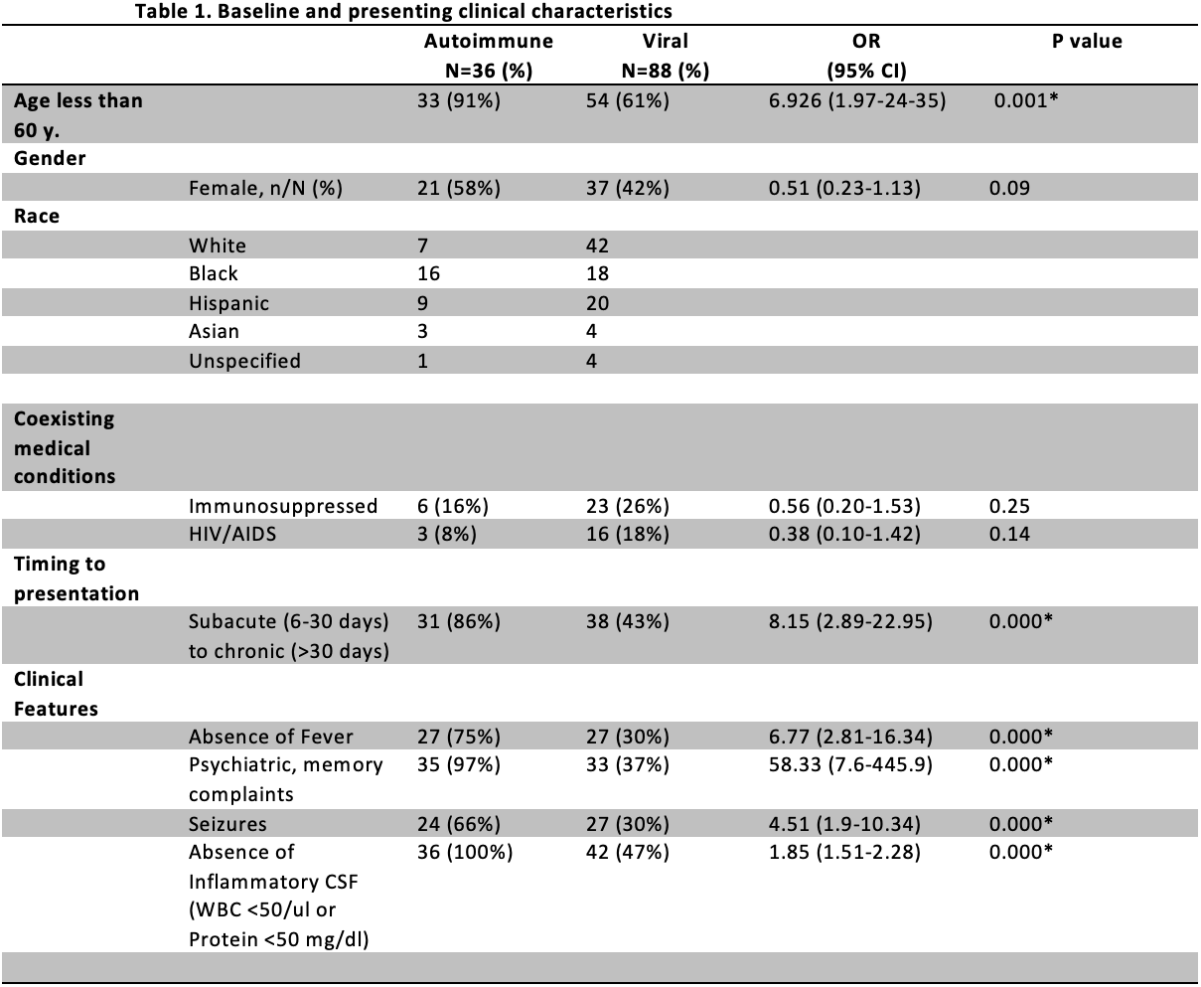

**Conclusion:**

Adults with encephalitis can be accurately stratified for the risk of having autoimmune encephalitis using clinical variables available upon presentation.

**Disclosures:**

**Rodrigo Hasbun, MD, MPH**, **Biofire** (Speaker's Bureau) **Rodrigo Hasbun, MD, MPH**, Biofire (Individual(s) Involved: Self): Consultant, Research Grant or Support

